# Patient autonomy in dentistry: demonstrating the role for shared decision making

**DOI:** 10.1186/s12911-020-01317-5

**Published:** 2020-12-02

**Authors:** Mareike Benecke, Jürgen Kasper, Christoph Heesen, Nina Schäffler, Daniel R. Reissmann

**Affiliations:** 1 Department of Prosthetic Dentistry, University Medical Center Hamburg-Eppendorf, Hamburg, Germany; 2 Department of Nursing and Health Promotion, Oslo Metropolitan University, Oslo, Norway; 3 Institute of Neuroimmunology and Clinical MS Research (INiMS), University Medical Center Hamburg-Eppendorf, Hamburg, Germany

**Keywords:** Patient preferences, Patient participation, Patient autonomy, Medical decision making, CPS, API

## Abstract

**Background:**

Evidence-based practice, decision aids, patient preferences and autonomy preferences (AP) play an important role in making decisions with the patient. They are crucial in the process of a shared decision making (SDM) and can be incorporated into quality criteria for patient involvement in health care. However, there are few studies on SDM and AP in the field of dentistry. This study explored patients’ autonomy preferences in dentistry in comparison to other medical domains, comparing them with patient preferences in two other cohorts of patients with different conditions and in different health care settings.

**Methods:**

A sample of 100 dental patients attending 16 dentists was consecutively recruited in a university-based prosthodontic clinic. Patients’ and dentists’ preferences regarding their roles in dental decision making for commonly performed diagnostic and treatment decisions were compared using the Control Preference Scale (CPS). This was followed by cross sectional surveys to study autonomy preferences in three additional cohorts recruited from general practices (n = 100), a multiple sclerosis clinic (n = 109), and a university-based prosthodontic clinic (n = 100). A questionnaire with combined items from the Autonomy Preference Index (API) to assess general and the CPS to assess specific preferences was used in the additional cohorts.

**Results:**

Dentists were less willing to give patients control than patients were willing to enact autonomy. However, decisions about management of tooth loss were considered relevant for a shared decision making by both parties. When comparing cohorts from different samples, the highest AP was expressed by people with multiple sclerosis and the lowest by patients in dentistry (means: dentistry 2.5, multiple sclerosis 2.1, general practice 2.4, *p* = .035). There were considerable intra-individual differences in autonomy preferences referring to different decision types (*p* < .001). In general, more autonomy was desired for treatment decisions in comparison to diagnostic decisions, for trivial compared to severe conditions, and for dental care compared to general practice (all: *p* < .001).

**Conclusion:**

There is an important role of patient participation in decision making in dentistry. Furthermore, PA should be considered with respect to specific medical decisions instead of assessing autonomy preferences in general implying a need for communication skills training of health care professionals.

## Background

Personal autonomy is widely valued. In the health care context, patient autonomy (PA) is a key concept in biomedical ethics. PA is usually associated with allowing or enabling patients to make their own decisions about which health care interventions they will or will not receive. Applied to medical decision making, PA is increasingly considered an important quality criterion in western countries. In medical decisions with a need to involve a health expert, PA becomes apparent in the patients’ participation in communication as described in the concept of shared decision making (SDM) [1]. PA has to be regarded for many reasons. From an ethical perspective, it is axiomatic to put every effort on supporting individuals’ freedom of will in decision making and in reaching consent on which intervention will or will not be performed, as reported in the guidance of the British General Medical Council [2]. From a scientific perspective, making best use of evidence for the individual patient is only possible when patient values are met. Therefore, PA is considered an essential part of evidence-based medicine (EBM). However, PA does not mean that clinicians should only provide different options and letting the patients make their decision alone. According to the concept of SDM, clinicians and patients work together to select the best option making use of best scientific evidence and considering clinicians’ clinical experience and patients’ preferences [3, 4]. From a clinical perspective, patients’ autonomous participation in their health management might improve health outcomes due to a better fit of health decisions with individual needs, leading also to higher compliance and satisfaction with decisions and outcomes [5].

Involving patients in medical decisions should not only be a practice in general medicine but be applied in dentistry too. Most of the medical decisions in dentistry consist of multiple options, each implying a specific set of patient relevant consequences and risks, e.g. there are several ways to manage tooth loss. Besides the option to do nothing and to live with a tooth gap, it might in an individual case be possible to use implants as a potentially long-term solution instead of a bridge, i.e. a conventional fixed dental prosthesis (FDP). A bridge does not require a series of invasive procedures but would affect the adjacent teeth. Basically, the decision will be made by considering and weighting advantages, disadvantages and risks, such as sustainability of dentures, invasiveness of the intervention, treatment and follow-up costs, and last but not least aesthetics [3]. Since patients’ individual experiences, expectations and emotions substantially matter to appraisal of the available options, this kind of decision is predestined for an approach that involves the patient.

Currently, knowledge regarding patients’ preferences for autonomy and desire for involvement in decision making in dental care is limited. While several of the available publications just provide a conceptual debate [3, 6–8], papers presenting empirical data [9–13] support the claim for more emphasis on patient autonomy and patient involvement in dentistry. While information preferences are consistently high, patient preferences to participate in decision making turn out lower [9, 10, 12, 13] but are however significant. Participation preferences are expressed also by older patients [11] and increase with severity of the treatment [12]. A model for the dentistry encounter provided by Sondell and Soderfeldt [14] indicates the importance of patient participation, however, without specifying quality of the information process. Little evidence is available on current habits, dentists’ attitudes and realization of patient involvement in dentistry [15]. Some evidence suggests that decision aids including evidence-based information are helpful tools to increase patient involvement [16–18] or patient satisfaction [19] or even cooperation and compliance [20]. However, detailed analyses of patient preferences regarding their autonomy in decision making in dentistry, a comparison with other medical populations, and the corresponding attitudes of the health care provider are still lacking.

This study explored patients’ autonomy preferences in dentistry. Dental patients’ preferences were compared with dentists’ attitude and in the context of other medical domains. The comparison aimed at identifying intra- and inter-individual variation of autonomy preference depending on type of decision and setting.

## Methods

### Subjects, study design, and setting

This cross-sectional study consists of three parts.

Firstly, a survey was carried out mapping the distribution of typical dentistry decisions at the Department of Prosthetic Dentistry, University Medical Center Hamburg-Eppendorf (UKE) in Hamburg, Germany over a 2-week period.

Secondly, the study surveyed patients’ autonomy preferences (PAP) in relation to the respective attitudes held by dentists regarding dental treatment decisions in the Department of Prosthetic Dentistry at the UKE. A consecutive sample of 100 patients and a full sample of all dentists of the clinic (n = 16) were recruited. To ensure maximum anonymity, names of patients and their respective dentist, sociodemographic as well as disease-specific data were not recorded.

Third, a cross sectional survey of PAP in dentistry, general practice (GP) and a multiple sclerosis (MS) outpatient unit was conducted by consecutively recruiting 100 patients in the waiting areas in each setting (N = 300 in total). Dental patients and patients with multiple sclerosis were contacted at the UKE, while patients in the primary care setting were recruited in three different GP offices, which were members of a primary medical care research network.

The study protocol (PV3452) was reviewed and approved by the Institutional Review Board of the Medical Association in Hamburg, Germany. Written informed consent was obtained from all participants prior to their enrollment. Participants and researchers received no monetary compensation.

### Measures

#### Medical decisions in dentistry

Decisions were documented by the dentists immediately after each appointment using a documentation sheet. This sheet was developed using a list of dental decisions, generated by a panel of two experienced dentists and an expert of methods, and underwent several evaluations to assure that the decision categories were both exhaustive and disjunctive. All dental decisions in the department were documented over a 2-weeks period to obtain an estimate of each decision’s frequency.

#### Autonomy preferences

PAP was assessed using two different methods, focusing on medical decision making in general and regarding specific medical problems. Using this approach allowed for the opportunity to compare these two methods. In addition to this, focusing on specific decisions also allowed for intra-individual comparisons of PAP.

#### The dental decision questionnaire

Based on the dental decisions identified in the first part of the study, a questionnaire with 15 items was developed to assess PAP. This item set comprised decisions referring to a wide range of dental diagnostics and treatments and covered the entire spectrum of dental care, except for orthodontics and oral surgery (Table 1).

**Table 1:** Dentists’ and patients’ ratings for patient autonomy in a set of dental decisions (DDQ with CPS as response scale). Lower scores indicate higher patient autonomy

No	Decisions (options)	Population		*P *value
		Dentists n = 16	Patients n = 100	
		*Mean (SD)*		
1	Whether to apply a local anesthesia (yes/no/wait)	2.5 (0.9)	1.8 (0.9)	.007*
2	Which anesthetic to use (common, adrenalin-reduced, adrenalin-free, superficial)	3.8 (0.5)	2.8 (0.9)	< .001*
3	Caries treatment (filling, crown, tooth extraction, temporary restoration, waiting)	2.6 (0.7)	2.3 (0.8)	.255
4	Treatment of painful dental nerve inflammation (endodontic treatment, apicoectomy, waiting)	2.6 (0.9)	2.6 (0.8)	.809
5	Treatment of gingivitis (prophylaxis, curettage, antibiotics, waiting)	3.1 (0.7)	2.4 (0.8)	.002*
6	Whether to extract a tooth (extraction, conservative treatment, waiting)	2.9 (1.0)	2.1 (0.9)	.006*
7	Problems with temporomandibular joint or masticatory muscles (physiotherapy, occlusal appliance, surgery, waiting)	2.5 (0.9)	2.4 (0.8)	.652
8	Complication with dentures, e.g. jiggling, fracture, sore spots (repair, lining, renewal, waiting)	2.3 (1.1)	2.0 (0.8)	.350
9	Initial tooth loss in the visible area—1–3 teeth missing (permanent / removable dentures, expanding existing dentures, dental implants, waiting)	1.9 (0.5)	1.9 (0.8)	.809
10	Initial tooth loss in the posterior area – 1–3 teeth are missing (permanent / removable dentures, expanding existing dentures, dental implants, waiting)	1.9 (0.5)	2.0 (0.8)	.749
11	Advanced tooth loss – only few teeth left (permanent / removable dentures, expanding existing dentures, dental implants, waiting	1.9 (0.5)	2.1 (0.7)	.556
12	Complete tooth loss (permanent / removable dentures, expanding existing dentures, dental implants, waiting)	1.9 (0.6)	2.0 (0.8)	.607
13	Diagnostic X-ray (dental film, orthopantomogram, no x-ray)	3.6 (0.7)	2.7 (0.9)	< .001*
14	Saliva test for caries risk assessment (yes/no)	2.8 (1.3)	2.3 (1.1)	.185
15	Prevention for teeth preservation (professional tooth cleaning [e.g. air-flow], scaling, individual prophylaxis [e.g. for children], periodontitis therapy)	2.7 (0.8)	1.9 (1.0)	.007*

The answering categories of the Control Preference Scale (CPS) [21] were used as the response scale for the items of this questionnaire. In the current study, the so called “pick-one” approach was applied providing an ordinal five-point response scale in a single item test evaluating a specific decision. The five steps range from “I want to make the decision alone” (0) to “I want the doctor to make the decision for me” (4).

The resulting Dental Decision Questionnaire (DDQ) was applied in the second part of our study to assess PAP. In addition, all participating dentists were asked to rate each decision on the list with regard to the decision’s sensitivity for patients’ preferences. To make this assessment, dental decisions were presented to participating dentists and patients as hypothetical cases.

#### The medical decision questionnaire

Based on bigger pool comprising several medical problems, ten were selected and corresponding items with response scales according to the CPS format were created. This selection covered three medical domains (4 items for GP, 3 items for dentistry, 3 items for MS) and provided variation between diagnostic vs. treatment, and serious vs. trifling decisions. To make this assessment, medical decisions were presented to participating patients as hypothetical cases.

This Medical Decision Questionnaire (MDQ) was applied in the third part of the study. With one exception, all patient groups (dental, general medicine, multiple sclerosis) were provided with the same set of items. Items specifically focusing on decisions in the field of multiple sclerosis were not given to dental or GP patients.

#### The autonomy preference inventory

The Autonomy Preference Inventory (API) [22] originally consists of six items prompting the patients to indicate their AP referring to medical decisions in general. The API presents statements indicating more or less autonomous attitudes exemplified by standard situations and provides for each statement a 5-point Likert scale ranging from “I completely agree” (0) to “I totally disagree” (4). However, since polarization of items varies, scores of items assessing preference for low autonomy were reversed to be in accordance with DDQ and MDQ response scale. Therefore, higher API scores represent lower patient autonomy. The API also provides a set of items assessing information needs. However, in our study just the six items addressing AP needs were applied. With regard to Cronbach’s alpha, the API scale turned out to show a much higher internal consistency when item 4 and 6 were excluded (6-item scale: alpha = .59; 4-item scale: alpha = .81). This finding is in line with previously published data of the API [22]. All analyses were, therefore, based on the four-item API. The API was applied only in the third part of the study.

### Statistical analyses

For analysis of PAP as assessed with API, DDQ, and MDQ, measures for central tendency (means) and variability (standard deviation; SD) were calculated for the entire population and subgroups considering the scores as quasi continuously scaled. Our statistical approach involved several steps corresponding to the three parts of the study.

Firstly, distribution of decisions in the studied department was analyzed descriptively by presenting frequencies and percentages.

Secondly, doctors’ and patients’ attitudes on whether and how much decisions should be shared were compared using unpaired *t*-tests for each of the 15 dental decisions in the DDQ.

Third, consistency of PAP within an individual and with respect to setting and type of medical decision was investigated using the scores of API and MDQ. Intra-individual consistency of PAP was approached by calculating Pearson correlations between API mean scores and each of the single MDQ items. Moreover, for each patient range between lowest and highest PAP as indicated for seven (dental and GP patients) or ten (MS patients) MDQ items, respectively, was calculated. In addition, intra-individual consistency in PAP was calculated as standard deviations of MDQ items within each patient and as intraclass correlation coefficient (ICC) based on an unifactorial analysis of variance (ANOVA) over the seven items that all patients filled in. This was followed by comparing PAP between patients of three different settings using ANOVA. For this analysis, means of all API items and the seven MDQ item all patients filled in were used. Finally, the impact of the specific type or character of a decision was studied using a repeated measurement design and the variants of decisions as within group factors in an ANOVA.

Alpha correction for multiple testing was performed using Bonferroni correction. To compensate for violation of sphericity, Greenhouse and Geisser corrections were used.

Missing values in the questionnaires were replaced by individual means for MDQ if only up to four of the seven items all patients filled in had missing information and for the API if for at least one of the four items a response was provided.

## Results

### Distribution of decisions in dentistry

Overall, 272 dental appointments and within these a total of 673 individual decisions (on average 2.5 per appointment) were documented (Table 1). Most frequently, dentists documented decisions about whether or not to use anesthesia for a treatment procedure (43%). Also, decisions about which anesthetic to use, treatment of caries, tooth extraction, problems with dentures or diagnostic radiology appeared in more than 25% of the appointments. Decisions on management of tooth loss or acutely inflamed nerves were made in about 10% of the appointments.

### Patients’ role preferences and dentists’ ratings on decisions’ sensitivity for individual preferences of dental decisions

In this second step of the study, all eligible patients and all doctors agreed to participate. When using the DDQ, dentists rated the decisions’ sensitivity for individual preferences on average over all assessed 15 dental decisions with a mean of 2.6 (SD 0.5). Patients indicated a role preference for the same selection of decisions with a mean of 2.1 (SD 0.5). This suggests, on average, dentists considered the patients’ participation in decision making less appropriate than the patients’ expressed preference (*p* = .008). This difference was statistically significant in 6 out of 15 decisions (Table 1). No differences were found to point into the opposite direction (patients desiring less autonomy than doctors would consider appropriate).

Both parties, however, allocated the locus of the decision slightly more on the doctor’s side, indicating more or less agreement about that dental decisions should mainly be made by the dentist rather than the patient. In contrast to most of the other decisions, those about managing tooth loss were considered relevant to patient involvement consistently by both parties (Table 1). Irrespective of progression stage or location of tooth loss in either the anterior (visible) or the posterior area of the mouth, all four decisions about tooth loss management were rated to be suitable for shared decision making by both dentists and patients.

### Intra-individual variance in patient autonomy preference

In the third part of the study, 97 of 104 eligible dentistry patients, 93 of 105 general medicine patients, and 109 of 110 patients with MS consented to participate. When using the MDQ, eleven percent of the patients used identical response option for all presented decisions (Table 2), indicating consistent PAP. 39% indicated PAP within presented decisions by using three adjacent response options within theoretical range of five options, representing some variance in PAP. 24% of the patients used a minimum of four or even exhausted the full theoretical range of five options, suggesting substantial intra-individual variance in PAP. Mean standard deviation of patient MDQ values covered 18% of the scale range (mean SD: 0.7; range: 0–4). Intraindividual consistency as assessed by the ICC based on the seven decisions relevant to all three cohorts was 0.27 suggesting high variability of decision-related PAP within individuals.

**Table 2:** Patients’ participation preferences related to general medicine, dentistry, and multiple sclerosis (MDQ with CPS as response scale) for all patients and stratified for setting. Lower scores indicate higher autonomy preference

	All n = 300	Population				*P* value
		General Medicine n = 93	Dentistry n = 97	Multiple sclerosis n = 109		
Decision type/medical domain		*Means (SD)*				
**General medicine**						
*Assume you have a sore throat, nasal congestion and cough for the last three days. Who should make the following decisions?*						
1	Whether to run an x-ray of your chest?	3.2 (0.8)	3.2 (0.8)	3.2 (0.7)	3.2 (0.8)	.900
2	Whether to treat your condition with cough syrup?	2.8 (1.0)	3.1 (0.9)	2.7 (1.1)	2.7 (1.0)	.018
*Assume that during routine examination a high blood pressure of 170/100 mmHg is measured. Who should make the following decisions?*						
3	Whether to undergo 24-h blood pressure monitoring?	3.5 (0.9)	3.4 (0.8)	3.5 (0.8)	3.5 (0.9)	.412
4	Whether to lower the blood pressure by use of drugs?	3.4 (0.8)	3.3 (0.8)	3.4 (0.8)	3.5 (0.9)	.144
Average over decisions in general medicine	3.2 (0.6)	3.3 (0.7)	3.2 (0.6)	3.1 (.6)	.273	
**Dentistry**						
*Assume you have lost 1 to 3 teeth in the visible range and meet the dentist. Who should make the following decisions?*						
5	Whether to use local anesthesia?	2.7 (1.0)	2.7 (1.0)	2.9 (0.9)	2.4 (1.0)	.002
6	Whether to apply diagnostic radiology?	3.6 (0.9)	3.6 (1.0)	3.7 (0.8)	3.5 (0.8)	.101
7	Whether and which treatment of your tooth loss is suitable?	2.7 (0.9)	2.7 (0.8)	2.8 (0.8)	2.7 (0.9)	.354
Average over decisions in dentistry	3.0 (0.7)	3.0 (0.7)	3.1 (0.6)	2.8 (0.7)	.010	
**Multiple sclerosis**						
*Who should make the following decisions on your chronic disease?*						
8	Whether and which immunotherapy to use as long-term treatment?	3.1 (0.8)	n/a	n/a	3.1 (0.8)	n/a
9	Whether to Magnet Resonance Imaging is needed?	3.5 (0.8)	n/a	n/a	3.5 (0.8)	n/a
10	Whether to use steroid treatment to manage an acute relapse?	3.2 (0.9)	n/a	n/a	3.2 (0.9)	n/a
Average over decisions in multiple sclerosis	3.2 (0.7)	n/a	n/a	3.2 (0.7)	n/a	
**Total**	3.1 (0.6)	3.1 (0.6)	3.2 (0.5)	3.1 (0.6)	.302	

Variability in patients’ reports was also reflected by medium or even low correlations between general PAP measured via API mean score and specific PAP measured via MDQ single items. Closest association between a PAP regarding a specific decision and API were shown for the decision whether to treat multiple sclerosis with immunotherapy (r = .43), while the decision whether to treat a cough with a syrup was not at all correlated with API (r = .08). Mean API and MDQ scores showed moderate correlation (r = .36).

### Patient autonomy preference and setting

General PAP assessed by the API differed substantially between the settings, with highest general PAP expressed by patients with MS and lowest by dental patients (API means: dentistry 2.5 [SD 1.1], MS 2.1 [SD 1.0], primary care 2.4 [SD 1.0]; *p* = .035).

A corresponding finding was observed for the level of specific decisions assessed using the MDQ (Table 2 and Appendix). While patient involvement in general medical decisions was considered equally relevant in all three settings, there were indeed differences between settings, especially in terms of dental medical decisions. Patients directly asked at the dental department were less willing to involve themselves into dental decisions than patients from the two other settings. Particularly, MS patients preferred to be involved in dental decisions more than dental patients, indicated by higher means in dental patients (3.1) than in MS patients (2.8) or in general medicine patients (3.0; *p* = .010; Table 2).

### Patient autonomy preference and type of a medical decision

According to the MDQ, analyses of decision-specific aspects revealed differences in levels of desired autonomy with regard to the type of medical decision, indicated by a statistically significant difference between the ten specific decisions (*p* < .001). Furthermore, PAP varied significantly between the three domains (MDQ means: general medical decisions 3.2; dental decisions 3.0, MS related decisions 3.2; *p* < .001; Table 2).

This also applied within the medical domain settings. Patients desired more autonomy in decisions about treatment (MDQ mean: 2.9) than about diagnostic measures (MDQ mean: 3.4; *p* < .001). Decisions about treatment of more severe conditions were associated with patients’ less willingness to overtake autonomy (MDQ mean: 3.1) compared to less severe conditions (MDQ mean: 2.7; *p* < .001).

## Discussion

This study provides insight into the relevance of autonomy preferences in dental settings, which so far has rarely been studied and, to the best of our knowledge, not been compared with other medical domains.

In the first part of the study, a high number of dental decisions were identified as of high relevance for shared decision making. Comparisons of the extent of PAP associated with different types of decisions such as decisions in varying medical domains are complicated due to potential confounders. Instead of artificially controlling or even standardizing sociodemographic variables in the three patient groups, we had strived to recruit representative cohorts. The findings of our study provide insight into both the importance of patient involvement in dentistry and the PAP-construct’s sensitivity towards several sources of variability. Dental patients desired to be involved into medical decisions that concern them. This preference is stronger than their dentists’ willingness to share decisions with their patients. In comparison to patients in other medical settings and to decisions related to other medical conditions, however, dental patients put less weight on involvement, in particular with regard to dental medical conditions, compared to patients in GP or in a chronic care setting. Dental patients seemed in general to claim slightly less autonomy compared to GP patients and chronic patients. With regard to the domain of the hypothetical decisions investigated, all groups indicated differential perception of autonomy needs. Highest PAP was expressed by MS patients, which was unsurprising because these patients likely already have broad experiences in medical decision making and have received high-end patient education as a result of managing their chronic condition. However, MS patients expressed highest PAP with regard to dental decisions. Not surprisingly, PAP was stronger in treatment than diagnostics and in less server than severe decision subjects. In addition, our data showed individual patients differentiated between decisions of varying kind when indicating their PAP. There is no one score fits all types of decisions. In contrary, the data showed considerable differences in range, extent, and variability of AP. In light of a mean correlation of r = .35 between the patients’ general and specific preferences, we have to realize that patient’s API score does not say much about this patient’s desire to be involved in a specific decision such as management of tooth loss or which immunotherapy to use. This finding might challenge the validity of the API scale or may indicate that findings from API and questionnaires using the CPS as response scale are somewhat related but obviously far from addressing the same construct. Our data show, however, considerable variability between decisions measured with the same response scale (CPS). Accordingly, the idea of a patient-specific invariant PAP should be challenged.

We are not aware of another study on PAP involving a comparable variety of measures, perspectives and decisions, which thus limits the possibility of comparing our findings with those of other studies. Our findings are in accordance with earlier studies on PAP in dentistry, indicating that the majority of patients prefer participating in the decision process, i.e. SDM [9, 10, 12, 13, 23]. Even though SDM was the preferred decision model amongst dental patients in our study, there is still a marginal majority preferring a passive role. This finding is consistent with findings from previous studies on dental patients as well as from other medical contexts [24]. Furthermore, variance of PAP with respect to setting and type of medical decision is also well documented [25, 26]. And finally, findings from our study are in line with conclusions from other studies, suggesting that findings from questionnaires such as API or those using CPI as a response scale are only of limited value to assess individual PAP with respect to a specific decision and at a specific occasion [27, 28].

We consider sample size (total N = 400) as a strength of the study as it ensures precision of the estimates and sufficient statistical power. On the other hand, the character of our study is clearly exploratory implying a couple of weaknesses that limit the final conclusiveness of our data. As we for practical reasons abstained from documentation of sociodemographic as well as disease-specific data, the study is lacking control of multiple potential cofounders known from the literature as important for PAP, such as age, education and medical conditions. However, we addressed cohorts naturally existing in the three example settings. Although our results regarding the impact of decision type are preliminary, they are clear enough to demonstrate importance of the decision type for the AP. In addition, our study is not representative for dentistry in general. We studied patients and decisions in a university-based prosthodontic clinic. In addition to this, when estimating preference sensitivity, we used a convenient sample of dentists of a prosthodontic clinic, which means a highly selective group in many regards. Data from e.g. resident dentistry might have given other results regarding group and setting. Methodological concerns might rise with regard to our assumption of PAP as a continuously scaled construct. It could be argued that the role distributions as given in the five CPS categories are distinctive qualities which hardly can be summarized, aggregated or correlated the way we did. However, several studies using the CPS considered the scale as quasi continuous and applied parametric statistical tests [23, 25, 26], as we did.

This study has important clinical implications. Decision support technologies should be developed according to existing guidelines to facilitate the patients’ involvement in decision making, to whatever extent they may prefer [29]. This could mean to develop decision specific decision aids for patients and using training for treatment providers in implementation of such technologies. Implementation of SDM most effectively works in combination of doctor- and patient-sided approaches [30]. Substantial inter- and intra-individual differences in PAP demonstrated in the current study and other studies [31] suggest that there is no strategy that fits all. In contrary, besides the more technical approaches the results imply the importance of generic communication skills as the key to individualization of care, particularly when it comes to making health decisions. Such doctor trainings already exist and have been tested in a dental setting, e.g. doktormitSDM [32, 33]. The training is specifically developed to enhance doctors’ responsiveness to varying extents of autonomy preference and proved efficient in increasing communication skills among dentists leading to stronger participation of dental patients in the decision-making process. Implementation of such trainings into both under- and postgraduate curricula is urgently needed to better meet patients’ individual preferences for participation in decision making and, as a consequence, provide better patient-oriented care [34].

## Conclusion

Based on the study’s findings it can be concluded that dental patients’ desire to be involved in decision making is comparable to other medical contexts. Furthermore, the study revealed also pronounced decision-type specificity of PAP, a finding which strongly challenges the construct of a general (implying consistent) PAP. The unique nature of individual preferences as reflected in the results the current study implies a strong need for development of communication skills in both under- and postgraduate medical training.

## Data Availability

All materials and data are available at the corresponding author.

## Appendix

**Table:**

Decision type / medical domain		Population														
		General medicine					Dentistry					Multiple sclerosis				
		%														
		0	1	2	3	4	0	1	2	3	4	0	1	2	3	4
General medicine																
*Assume you have a sore throat, nasal congestion and cough for the last three days. Who should make the following decisions?*																
1	Whether to run an x-ray of your chest?	4.7	4.7	58.8	27.1	4.7	2.1	5.2	65.6	21.9	5.2	4.8	5.7	63.8	18.1	7.6
2	Whether to treat your condition with cough syrup?	7.0	10.5	54.7	24.4	3.5	15.5	22.7	43.3	12.4	6.2	15.4	15.4	54.8	11.5	2.9
*Assume that during routine examination a high blood pressure of 170/100 mmHg is measured. Who should make the following decisions?*																
3	Whether to undergo 24-h blood pressure monitoring?	1.2	7.2	48.2	28.9	14.5	2.1	1.0	57.3	22.9	16.7	2.9	2.9	57.1	29.5	7.6
4	Whether to lower the blood pressure by use of drugs?	1.2	3.6	51.2	29.8	14.3	1.0	6.2	54.6	24.7	13.4	2.9	1.0	67.6	21.0	7.6
Dentistry																
*Assume you have lost 1 to 3 teeth in the visible range and meet the dentist. Who should make the following decisions?*																
5	Whether to use local anesthesia?	12.9	21.2	51.8	9.4	4.7	8.3	13.5	64.6	9.4	4.2	22.1	27.9	40.4	6.7	2.9
6	Whether to apply diagnostic radiology?	3.5	3.5	44.7	30.6	17.7	0.0	2.1	41.1	41.1	15.8	1.9	4.8	52.4	28.6	12.4
7	Whether and which treatment of your tooth loss is suitable?	11.9	20.2	58.3	8.3	1.2	10.3	7.2	73.2	8.3	1.0	14.3	18.1	56.2	8.6	2.9
Multiple sclerosis																
*Who should make the following decisions on your chronic disease?*																
8	Whether and which immunotherapy to use as long-term treatment?	–	–	–	–	–	–	–	–	–	–	3.9	7.8	72.8	9.7	5.8
9	Whether to Magnet Resonance Imaging is needed?	–	–	–	–	–	–	–	–	–	–	1.0	4.9	52.4	29.1	12.6
10	Whether to use steroid treatment to manage an acute relapse?	–	–	–	–	–	–	–	–	–	–	6.8	7.8	60.2	15.5	9.7

0: “I want to make the decision alone”

1: “Mainly I want to make the decision after considering the doctor’s opinion”

2: “Doctor and I should make the decision together”

3: “Mainly doctor should make the decision after considering your opinion”

4: “I want the doctor to make the decision for me”

## Abbreviations

PA: Patient autonomy
AP: Autonomy preference
PAP: Patient autonomy preferences
SDM: Shared decision-making
EBM: Evidence based medicine
EBD: Evidence based dentistry
CPS: Control preference scale
API: Autonomy preference inventory
DDQ: Dental decision questionnaire
MDQ: Medical decision questionnaire
GP: General practitioner
MS: Multiple sclerosis
